# P-469. Epidemiology of invasive fungal disease in children with relapsed or refractory leukemia

**DOI:** 10.1093/ofid/ofaf695.684

**Published:** 2026-01-11

**Authors:** Caitlin N Brammer, Soumya Jaiswal, Benhur S Cetin, Hilary Miller-Handley, Mark Murphy, Caroline Maguire, Grant C Paulsen, Lara A Danziger-Isakov, William R Otto

**Affiliations:** Cincinnati Children's Hospital Medical Center, Cincinnati, OH; University of Cincinnati College of Medicine, Mason, Ohio; Cincinnati Children's Hospital Medical Center, Cincinnati, OH; Cincinnati Children's Hospital Medical Center, Cincinnati, OH; Cincinnati Children's Hospital Medical Center, Cincinnati, OH; Cincinnati Children's Hospital Medical Center, Cincinnati, OH; Cincinnati Children's Hospital Medical Center, Cincinnati, OH; Cincinnati Children's Hospital, Cincinnati, OH; Cincinnati Children's Hospital Medical Center, Cincinnati, OH

## Abstract

**Background:**

Advances in chemotherapeutic treatment have led to improvements in survival for children with relapsed leukemia. Despite this progress, treatment related mortality remains high, in particular that due to infectious diseases. Invasive fungal disease (IFD) is a significant cause of morbidity and mortality for children with relapsed or refractory leukemia, but there remains limited data about the epidemiology of IFD in these high-risk patients. This study sought to define the epidemiology of IFD for children with relapsed leukemia.

**Methods:**

This retrospective study included children and young adults treated for relapsed or refractory leukemia at Cincinnati Children’s Hospital Medical Center between 2015-2023. Micafungin was standard antifungal prophylaxis for the majority of this time period. Demographic, clinical, and microbiological data were abstracted, including positive mycological testing. Cases of IFD were defined using the EORTC/MSG consensus definitions. Incidence of IFD was calculated. Descriptive statistics summarized the diagnostic evaluation at each site.

**Results:**

The cohort included a total of 190 patients who contributed 605 chemotherapy courses. Twenty patients (20/190, 10.5%, 95% CI 6.5%-15.8%) developed 21 distinct episodes of IFD. There were 18 cases of proven IFD and 3 cases of probable IFD. Cases were split evenly between yeasts (n=10) and molds (n=11). The most common genus identified was Candida spp, followed by Aspergillus spp, though nearly half of all mold infections were caused by non-Aspergillus, non-Mucorales molds. Of infections involving yeasts, 8/10 presented as fungemia while the remaining 2/10 were disseminated infections. The majority of mold infections occurred in the sinuses (n=5) or lungs (n=4), with two cases of cutaneous disease that occurred after trauma.

**Conclusion:**

In this single center cohort of children and young adults with relapsed or refractory leukemia, IFD was common despite widespread antifungal prophylaxis use. The incidence of IFD in this study was higher than that of contemporary cohorts with acute leukemia. Mold infections occurred more commonly than invasive candidiasis or other infections with yeasts. Further research is needed to identify additional risk factors for IFD in this population.

**Disclosures:**

Grant C. Paulsen, MD, Moderna, Inc: Grant/Research Support|Pfizer: Grant/Research Support|Sanofi: Grant/Research Support Lara A. Danziger-Isakov, MD, MPH, Aicuris: Grant/Research Support|Ansun BioPharma: Grant/Research Support|Astellas: Advisor/Consultant|Astellas: Grant/Research Support|Merck: Advisor/Consultant|Merck: Grant/Research Support|Pfizer (Any division): Grant/Research Support|Takeda: Grant/Research Support

